# Alcohol Consumption among HIV-Infected Persons in a Large Urban HIV Clinic in Kampala Uganda: A Constellation of Harmful Behaviors

**DOI:** 10.1371/journal.pone.0126236

**Published:** 2015-05-11

**Authors:** Bonnie Wandera, Nazarius Mbona Tumwesigye, Joaniter Immaculate Nankabirwa, Andrew Ddungu Kambugu, Rosalind Parkes-Ratanshi, David Kaawa Mafigiri, Saidi Kapiga, Ajay K. Sethi

**Affiliations:** 1 Department of Epidemiology and Biostatistics, Makerere University School of Public Health, Kampala, Uganda; 2 Infectious Diseases Institute, Makerere University College of Health Sciences, Kampala, Uganda; 3 Department of Medicine, Makerere University College of Health Sciences, Kampala, Uganda; 4 Department of Social Work and Social administration, Makerere University College of Humanities and Social Sciences, Kampala, Uganda; 5 Department of Infectious Disease Epidemiology, London School of Hygiene & Tropical Medicine and Mwanza Interventional Trials Unit, Mwanza, Tanzania; 6 Department of Population Health Sciences, University of Wisconsin-Madison, Madison, Wisconsin, United States of America; Massachusetts General Hospital, UNITED STATES

## Abstract

**Introduction:**

Alcohol use by persons living with HIV/AIDS (PLWHA) negatively impacts the public health benefits of antiretroviral therapy (ART). Using a standardized alcohol assessment tool, we estimate the prevalence of alcohol use, identify associated factors, and test the association of alcohol misuse with sexual risk behaviors among PLWHA in Uganda.

**Methods:**

A cross-section of PLWHA in Kampala were interviewed regarding their sexual behavior and self-reported alcohol consumption in the previous 6 months. Alcohol use was assessed using the alcohol use disorders identification test (AUDIT). Gender-stratified log binomial regression analyses were used to identify independent factors associated with alcohol misuse and to test whether alcohol misuse was associated with risky sexual behaviors.

**Results:**

Of the 725 subjects enrolled, 235 (33%) reported any alcohol use and 135 (18.6%) reported alcohol misuse, while 38 (5.2%) drank hazardous levels of alcohol. Alcohol misuse was more likely among subjects not yet on ART (adjusted prevalence ratio [aPR] was 1.65 *p*=0.043 for males and 1.79, *p*=0.019 for females) and those with self-reported poor adherence (aPR for males=1.56, *p*=0.052, and for females=1.93, *p*=0.0189). Belonging to Pentecostal or Muslim religious denominations was protective against alcohol misuse compared to belonging to Anglican and Catholic denominations in both sexes (aPR=0.11 for men, *p*<0.001, and aPR=0.32 for women, *p*=0.003). Alcohol misuse was independently associated with reporting risky sexual behaviors (aPR=1.67; 95% CI: 1.07–2.60, *p*=0.023) among males, but not significant among females (aPR=1.29; 95% CI: 0.95–1.74, *p*=0.098). Non-disclosure of HIV positive status to sexual partner was significantly associated with risky sex in both males (aPR=1.69; *p*=0.014) and females (aPR 2.45; *p*<0.001).

**Conclusion:**

Alcohol use among PLWHA was high, and was associated with self-reported medication non-adherence, non-disclosure of HIV positive status to sexual partner(s), and risky sexual behaviors among male subjects. Interventions targeting alcohol use and the associated negative behaviors should be tested in this setting.

## Introduction

Increased availability of antiretroviral therapy (ART) in sub-Saharan Africa (SSA) has saved an estimated 9 million life years [1] with more benefits anticipated as new World Health Organization (WHO) guidelines for earlier initiation of ART get fully implemented [2,3].

The public health benefits of ART are dependent on subjects’ adherence to prescribed treatment and adopting behaviors that limit further transmission of HIV from persons living with HIV/AIDS (PLWHA), among a host of other individual, social and health system factors [4–6]. Recent studies have reported high adherence to ART among PLWHA, reduction in number of sexual partners, and increased condom use after both HIV counseling and testing and after initiation of ART [7–10]. However, some patients continue to engage in risky behaviors, that could lead to further HIV transmission [11]. PLWHA who report using alcohol have an increased likelihood of engaging in risky sexual activities [12], including unprotected sexual intercourse [13–16]. This is particularly problematic in the SSA region where HIV transmission is mainly through the heterosexual transmission route [1]. A meta-analysis by Hendershot and colleagues showed that subjects using alcohol were twice likely to report poor adherence to ART as compared to abstainers [17]. Kalichman and colleagues demonstrated that more than half of PLWHA using alcohol were still potentially infectious based on their plasma HIV viral loads despite being on combination ART [18].

Uganda has one of the highest per capita alcohol consumption levels in SSA [19,20] and frequent heavy episodic drinking is common among Ugandans [21]. Understanding the burden of alcohol use and the associated sexual risk behaviors among PLWHA in the region is essential in designing locally appropriate interventions to reduce alcohol consumption that could potentially lead to reduced HIV transmission risk behaviors. Published studies on alcohol use among PLWHA in SSA are limited by small study populations and do not use standardized alcohol assessments tools, as highlighted in the review by Woolf-King and colleagues [22]. In order to address these limitations, we conducted a large study to estimate the prevalence of any alcohol use, misuse, and hazardous consumption among HIV-infected patients receiving care and treatment at a large HIV clinic in Kampala, Uganda. Additionally the study delineates factors associated with reported alcohol use, and tests whether reported alcohol misuse is independently associated with risky sexual behaviors in this population.

## Methods

### Study design, setting and population

A cross sectional study was conducted between October 2012 and May 2013 among PLWHA attending the adult Infectious Diseases Institute (IDI) clinic located within the Mulago National Referral and Teaching Hospital campus in Kampala City. This outpatient clinic provides comprehensive HIV prevention, care and treatment at no cost to a total of 10,000 patients with daily clinic visits averaging 180 patients per day. The clinic provides routine group and individual counseling services to all new patients, and thereafter, when needed or when prescribed by the treating clinician. Counseling services include general supportive counseling for positive living, adherence to clinic appointments and prophylaxis treatments, intensive pre-ART counseling, HIV disclosure, reducing risky sexual behaviors, alcohol and drug use, and adherence support services. The clinic runs for eight hours per day from Monday to Friday.

All patients presenting to the clinic were registered upon arrival; we systematically sampled every 15th patient arriving at the clinic and assessed their study eligibility. Patients were included into the study if they were i) aged ≥18 years; ii) capable of answering interview questions as assessed by a Karnofsky clinical performance score >50%; iii) willing to provide written informed consent for study participation; and iv)self-reported as not pregnant, if female.

### Data collection

At study enrollment, a structured questionnaire was administered by trained nurse counselors in English or Luganda (the native and most commonly used local language in the clinic catchment region). Demographic characteristics including age, gender, religion, number of children ever born, individual income in the last month, highest educational level attained, and employment status were collected. In addition, information on alcohol consumption, sexual behaviors within the last six months, adherence to prescribed treatment (ART or co-trimoxazole for those not yet on ART) and clinical data was collected. Data on alcohol consumption were collected using the AUDIT questionnaire and additional information on actual days on which alcohol consumption occurred within the past 30 days was collected using the Alcohol Timeline Follow-back method [23]. The AUDIT is an internationally-validated tool for screening alcohol consumption in the recent past (up to 1 year) for use in both clinical and community settings [24]. The AUDIT is a 10-item questionnaire with 8 questions containing 5 response categories and 2 questions having 3 response categories with possible scores of 0–4 for each question. The 10 items consist of 3 questions that assess hazardous alcohol use, 3 questions on dependence symptoms and 4 questions on the harmful alcohol use domain. The sum of the scores from the 10 questions yields the final AUDIT score ranging from 0–40 with higher scores correlated to having an alcohol use disorder. The first 3 items of the AUDIT, which are related to hazardous alcohol consumption (AUDIT-C), have been used separately to screen for alcohol misuse in primary care settings [25]. The AUDIT-C component has a maximum score of 12 points and minimum score of 0 points. Information on the amount of money spent on a typical drinking day in the last 6 months was also collected.

Information collected on sexual behavior was captured once but encompassed a summary of sexual behavior within the last 6 months and included, marital status, engaging in sexual intercourse, total number of sexual partners, condom use at last sexual encounter, consistent condom use at all sexual encounters within the last 6 months, use of alcohol in sexual contexts, having sex while drunk and HIV sero-status of sexual partner(s).

Clinical data were abstracted from subjects’ clinic records; specifically, the WHO HIV clinical stage, nadir and most recent (within last 12 months) CD4+ cell count, and receipt of ART and/or co-trimoxazole. Self-reported adherence was assessed in both ART and non-ART receiving subjects. Among subjects on ART, adherence was based on subjective ranking on a visual analogue scale (VAS) of all antiretroviral drugs taken out of all antiretroviral drugs prescribed within the last 3 months. Among subjects not yet on ART, the VAS was based on co-trimoxazole tablets taken out of all co-trimoxazole prescribed in the last 3 months. All subjects registered in the clinic receive co-trimoxazole (or dapsone) as prophylaxis against respiratory and diarrheal infections that is taken daily even after initiating ART. Depression level was scored using the 10-item Center for Epidemiology Studies on Depression tool (CESD-10), which has been validated in sub-Saharan Africa [26].

### Data management and analysis

All data were double entered in a customized Microsoft Access (Redmond, WA, USA) database and exported to SAS V 9.1.3 (SAS Inst., Carry, NC, USA) for statistical analysis.

Analyses were stratified by patient gender due to previously known gender differences in alcohol use and sexual risk behaviors in sub-Saharan Africa [22,27]. Descriptive continuous variables were summarized as medians and interquartile ranges while categorical variables were summarized as percentages.

The outcome of interest, alcohol consumption, was examined as: 1) any alcohol use, 2) alcohol misuse, and 3) hazardous alcohol consumption. The operating definition for any alcohol use was any reported alcohol consumption of any amount at least once in the previous 6 months; alcohol misuse was defined as scoring 3 points or higher on the AUDIT-C tool while hazardous alcohol consumption was defined scoring 8 or more points on the full AUDIT [28,29].

A subject was considered sexually active if they reported engaging in sexual intercourse at least once within the last 6 months. The majority of sexual relationships in this region are reportedly heterosexual, and there was no explicit inquiry on gender of sexual partner(s). Sexual behavior outcomes of interest in the preceding 6 months were classified as risky sex and high-risk sex. Risky sex was defined as reporting either unprotected sexual intercourse with a partner of unknown HIV sero-status or known HIV negative, or reporting sexual intercourse with 2 or more sexual partners irrespective of their HIV sero-status. High-risk sex was defined as reporting both behaviors in the previous 6 months.

Reported alcohol misuse and risky sex were common in the study population. Thus, we examined how these behaviors were associated with factors of interest using log binomial regression. This approach yields the prevalence ratio (PR), which is a better approximation of relative risk than the odds ratio in a cross-sectional study when the prevalence of the outcome of interest is not rare (i.e., >10% of the population), as was the case with both alcohol misuse and risky sexual behavior. The odds ratio tends to overestimate the risk ratio in this context, and distorts interpretation of the strength of association [30].

To identify independent factors associated with alcohol misuse, univariable and multivariable log binomial regression models were built to estimate the relative prevalence of alcohol misuse by the independent variables of interest. During the iterative modeling process some transitional models failed to converge due to numerical instability of the log binomial models, we applied the modified poisson model with robust variance estimates [31]. We constructed univariable models with alcohol misuse as the dependent variable and examined its association with socio-demographic, behavioral, and clinical factors. Unadjusted PR (uPR) and their corresponding 95% confidence intervals (CI) and *p*-values were estimated. All factors from univariable analyses with probability values of 0.2 or less were candidates for inclusion in multivariable models. Among males, the initial full multivariable model included age, religion, WHO stage of HIV disease, CD4 cell count, ART receipt and adherence to ART or co-trimoxazole. For females, factors fit into the initial full multivariable model were age, religion, ART receipt status, HIV diseases stage CESD score category, and adherence to ART or co-trimoxazole. The modeling process involved a rational iterative removal of factors from a full multivariable model provided the removal did not result in a significant change in the overall model fit as assessed by both the quasi-likelihood information criterion (QIC-U) and/or was conceptually important and/or biologically plausible. To identify statistically significant factors associated with alcohol misuse, the removal process was repeated until no further covariate was eligible for removal. Factors contained in final multivariable models were ART receipt status, religion, and medication adherence and CD4 cell count among males while final models in females contained ART receipt status, religion, and medication adherence and CESD score. The final results are presented as adjusted prevalence ratios (aPR), 95% CIs and corresponding p-values.

To assess whether alcohol misuse was independently associated with reporting any risk sexual behaviors, gender-specific log binomial regression models with risky sex as the outcome and alcohol misuse as the independent predictor were constructed. The iterative modeling procedures described above was carried for this analysis as well.

### Ethics Statement

There was no monetary compensation or incentive provided to subjects for study enrolment. The study was approved by the scientific and ethical review boards of IDI, Makerere University School of Public Health Research and Ethics Committee and the Uganda National Council of Science and Technology.

## Results

A total of 755 individuals were screened of which 725 (96%) were enrolled. Among the 30 subjects not enrolled, 22 were ineligible, while the remaining 8 mostly reported a lack of time to answer the interview questions.

Descriptive characteristics (Table 1), show the median (interquartile range; IQR) age of participants was 39 (31, 46) years. The majority, 452 (61.3%), were female and 319 (44.0%) were married/cohabiting while 283(39.0%) were separated/divorced. The majority (88.3%) reported some form of employment; data on individual income in the last month was reported by 94% of subjects. Overall, 441/682, 64.7%, earned between 20–400 United States Dollars (USD) in the previous month. Over 80% (n = 597) were receiving ART and 417 (57.5%) were diagnosed as having WHO stage of HIV disease III & IV. The median (IQR) CD4 cell count at the time of interview was 362 (248, 537) cells/μl. Plasma viral loads are not routinely done at the clinic and were not available for this study.

**Table 1:** Characteristics and prevalence of alcohol consumption among 725 HIV-infected men and women attending the Infectious Diseases Institute HIV Clinic, Kampala, Uganda.

Characteristic	Total subjects enrolled		Subjects who reported alcohol consumption at least once in the last 6 months	
	N	%	N	%
Sex				
Male	273	37.7	112	41.0
Female	452	62.3	127	28.1
Receiving ART				
Yes	597	82.3	183	30.7
No	128	17.7	56	43.8
Median (IQR) age	39	31–46	38	32–45
Marital status				
Never married	123	17.0	37	30.1
Married or cohabiting	319	44.0	111	34.8
Separated or widowed	283	39.0	91	32.2
Education level				
Primary or less	364	50.2	116	31.9
Secondary level	298	41.1	98	32.9
Tertiary level/diploma	63	8.7	25	39.7
Religion				
Roman Catholic	259	35.7	111	42.9
Anglican	238	32.8	103	43.3
Pentecostal Christianity, Islam, or Seventh Day Adventism	228	31.5	25	11.0
Paid employment^1^				
Yes	640	88.3	214	33.4
No	85	11.7	25	29.4
Income in last month				
< 20 USD	221	30.5	63	28.5
20–400 USD	441	60.8	155	35.1
> 400 USD	20	2.8	9	45.0
Declined	43	5.9	12	27.9
Number of children ever born				
0	73	10.1	20	27.4
1 to 4	404	55.7	138	34.2
>4	248	34.2	81	32.7
HIV clinical stage				
I or II	308	42.5	106	34.4
III or IV	417	57.5	133	31.9
Median (IQR) most recent CD4 cell count (cells/mm3)	362	248–537	385	255–544
Median (IQR) nadir CD4 cell count (cells/mm3)	163	58–293	175	72–335
History of TB treatment				
Yes	162	22.4	56	34.6
No	560	77.6	182	32.5
CESD-10 depression score				
< 10 points	486	67.0	171	35.2
≥ 10 points	239	33.0	68	28.5
Sexually active in last 6 months				
Yes	479	67.2	190	39.7
No	234	32.8	47	20.1
Unprotected sex in last 6 months^2^				
Yes	132	18.2	57	43.2
No	593	81.8	182	30.7
Reported 2 or more sex partners				
Yes	115	17.3	58	50.4
No	551	82.7	168	30.5
Reported any risk sex				
Yes	197	27.2	92	46.7
No	528	72.8	147	27.8
Reported high risk sex				
Yes	39	5.4	22	56.4
No	686	94.6	217	31.6

^1^ Housewives without other work were classified as unemployed.

^2^ Defined as sex without condom with a partner who is HIV negative or without unknown HIV status.

Only 37 of 683 (4.7%) reported current cigarette smoking and only 17 subjects reported recreational drug use, almost all of them reporting smoking marijuana.

### Alcohol consumption in the preceding 6 months

**Fig 1:**
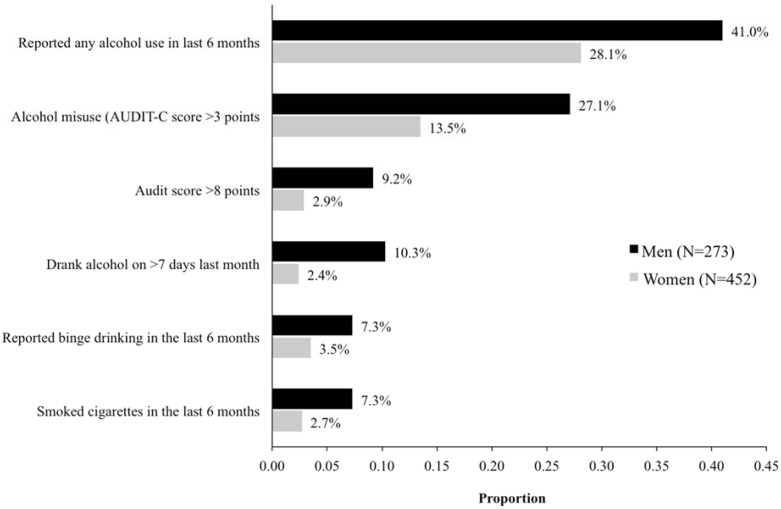
Proportions of men and women reporting different alcohol and smoking outcomes among 725 HIV-infected individuals attending the Infectious Disease Institute HIV Clinic, Kampala, Uganda.

Overall, 239/725 (33.0%) subjects reported consuming alcohol at least once during the preceding 6 months. Males were more likely to have consumed alcohol than females (41% versus 28.1%, *p*<0.001). Proportions of different alcohol use outcomes by gender are presented in Fig 1 and all show that males reported higher alcohol use outcomes than females. Almost a quarter (175/725, 24.1%) of participants reported alcohol consumption in the previous month with males significantly more likely than females to do so (33.7% vs. 18.4%; *p*<0.001). Among 239 subjects who reported any alcohol consumption within the last 6 months, 135 (56.5%) scored 3 or more points on the AUDIT-C and were categorized as misusing alcohol, representing 18.6% of the total study sample. Similarly, males (66.1%) were more likely than females (48.0%) to misuse alcohol (*p*<0.001). Among subjects reporting any alcohol use, 36 (15.1%) reported drinking 6 or more units of alcohol at one sitting within the last 6 months. Only 16% (38/239) of subjects who drank scored ≥ 8 points on the full AUDIT and were classified as hazardous drinkers, representing 5.2% of the total study sample. Again, among drinkers, males were significantly more likely than females to be hazardous drinkers (22.3% vs. 10.2%, *p*<0.001). On average, 2.31 (standard deviation; SD = 1.9) USD were spent directly on alcohol on a typical drinking day. The average cost of most beer brands at the local drinking venues with in the last year was approximately 1.10 USD.

### Factors associated with alcohol misuse among PLWHA

In the final multivariable model (Table 2), among males, the prevalence of alcohol misuse was 65% more likely among subjects not taking ART compared to those taking ART (*p* = 0.043) and 56% more likely among those with less than perfect adherence on medication (*p* = 0.052). Compared to Anglican religious faith, Pentecostal/Muslim, faiths that openly and proactively prohibit alcohol consumption, was associated with an 89% lower prevalence of alcohol misuse (*p*<0.001). There was no statistical difference in the prevalence of alcohol misuse between Anglican and Roman Catholic denominations. Monthly income, age, history of tuberculosis treatment and current CD4 cell counts were not independently associated with reporting alcohol misuse.

**Table 2:** Factors associated with alcohol misuse (AUDIT-C ≥ 3) among 74 HIV-infected men and 61 HIV-infected women attending the Infectious Disease Institute HIV Clinic, Kampala, Uganda.

Characteristic	Males			Females		
	AUDIT-C ≥3N (%)	Unadjusted PR(95% CI)	Adjusted PR(95% CI)	AUDIT-C ≥3N (%)	Unadjusted PR (95% CI)	Adjusted PR(95% CI)
Receiving ART						
Yes	61 (82.4)	1.0	1.0	40 (65.6)	1.0	1.0
No	13 (17.6)	1.32 (0.80–2.20)	1.65 (1.02–2.67)*	21 (34.4)	2.08 (1.29–3.34)**	1.79 (1.10–2.91)*
Mean (SD) Age (per 10 years)	42.8 (11.1)	1.17 (0.95–1.43)		34.6 (18.4)	0.80 (0.65–0.99)*	0.82 (0.68–0.99)*
Marital status						
Never married	12 (16.2)	1.11 (0.64–1.91)		12 (19.7)	1.05 (0.54–2.06)	
Married or cohabiting	49 (66.2)	1.0		19 (31.2)	1.0	
Separated/widowed	13 (17.6)	0.73 (0.43–1.26)		30 (49.2)	1.04 (0.61–1.79)	
Education level						
Primary or less	29 (39.0)	1.06 (0.69–1.63)		37 (60.7)	1.14 (0.69–1.87)	
Secondary	34 (46.0)	1.0		21 (34.4)	1.0	
Tertiary/diploma	11 (15.0)	1.22 (0.69–2.17)		3 (4.9)	0.85 (0.27–2.65)	
Paid employment						
Yes	69 (93.2)	0.86 (0.40–1.82)		54 (88.5)	1.37 (0.65–2.88)	
No	5 (6.8)	1.0		7 (11.5)	1.0	
Income in last month						
<20 USD	9 (12.2)	0.79 (0.42–1.46)		25(41.0)	1.12 (0.68–1.83)	
20–400 USD	56 (75.7)	1.0		30 (49.2)	1.0	
>400 USD	9 (12.2)	1.01 (0.56–1.84)		6 (9.8)	1.61 (0.73–3.54)	
Religion						
Anglican	38 (51.4)	1.0	1.0	22 (36.1)	1.0	1.0
Roman Catholic	33 (44.6)	0.75 (0.51–1.10)	0.75 (0.51–1.09)	31 (50.8)	1.33 (0.81–2.19)	1.28 (0.77–2.11)
Other religions ^1^	3 (4.0)	0.11 (0.04–0.36)**	0.11 (0.03–0.33)**	8 (13.1)	0.32 (0.15–0.71)*	0.32 (0.15–0.69)*
WHO/HIV Clinical Stage						
I & II	27 (36.5)	1.39 (0.93–2.07)		40 (65.6)	1.86 (1.14–3.06)*	
III & IV	47 (63.5)	1.0		21 (34.4)	1.0	
Mean (SD) CD4 cell count(per 100 cells/mm^3^)	328.7 (162.8)	0.93 (0.86–1.02)	1.00 (0.99–1.00)	488.3 (254.8)	1.06 (0.99–1.14)	
Mean (SD) nadir CD4 cell count(per 100 cells/mm^3^)	177.2 (167.2)	1.05 (0.94–1.17)		284.4 (257.0)	1.08 (0.99–1.17)	
Self-reported adherence to medication in the last 3 months^2^						
Adherent	57 (77.0)	1.0	1.0	48 (78.7)	1.0	1.0
Non-adherent	17 (23.0)	1.44 (0.92–2.27)	1.56 (1.00–2.44)	13 (21.3)	2.12 (1.24–3.64)*	2.36 (1.34–4.14)*
CESD-10 Depression Score^3^						
<10 points	53 (71.6)	1.08 (0.70–1.67)		46 (75.4)	1.62 (0.94–2.81)	1.93 (1.12–3.34)*
≥10 points	21 (28.4)	1.0		15 (24.6)	1.0	1.0

* *p*<0.05;

** *p*<0.001

^1^ Refers to Pentecostal Christianity, Islam, or Seventh Day Adventism.

^2^Medications included ART and/or co-trimoxazole. Adherent implied >95% score on a Visual Analogue Scale.

^3^Based on the10-item Center for Epidemiologic Studies on Depression scale.

Among females, younger individuals, those not receiving ART, reporting less than perfect adherence to medication and those with <10 score on the CESD depression scale were on average twice likely to report alcohol misuse (Table 2). As was observed in males, belonging to Pentecostal or Muslim faith was protective of alcohol misuse as compared to Anglican and Catholic denominations.

### Reported sexual behavior

Among all the 725 subjects interviewed, 479 (67.2%) reported sexual intercourse within the last 6 months. The majority, (403/479, 84.3%) reported their last sexual encounter was with their spouse or long-term acquaintance while the remaining 15.7% reported their last sexual encounter was with a casual sexual partner.

Of the 479 sexually active subjects, 282 (59.8%) reported using a condom at the last sexual encounter while 205/479 (43.4%) reported consistent condom use with in the last 6 months. Most who reported protected sex used male condoms; only two subjects reported using female condoms. Among the sexually active subjects, 23.2% (107/479) reported sexual intercourse with 2 or more sexual partners within the last six months. A high proportion (73.4%) of sexually active subjects had disclosed their HIV status to their main sexual partner. The main sexual partners of the sexually active subjects were mostly HIV positive (n = 219), but many were HIV negative (n = 104) of unknown/undisclosed HIV sero-status (N = 151). Thirty-nine (5.4%) individuals reported high-risk sex within the last six months.

### Association between alcohol consumption and risky sexual behaviors

Of the 479 sexually active subjects, 9.2% (54/479) reported engaging in sexual activity while taking alcohol, of which 17 (31.5%) were drunk or intoxicated at that last sexual encounter.

Selected sexual behaviors stratified by reported alcohol use in the last 6 months are shown in Table 1. Without considering gender differences, subjects reporting any alcohol use were more likely to report sexual intercourse (uPR = 1.98; 95% CI: 1.49–2.61), two or more sexual partners, (uPR = 1.98; 95% CI: 1.43–2.75), unprotected sex with HIV negative/unknown sero-status partner (uPR = 1.54; 95% CI: 1.14–2.10), and high risk sex (uPR = 2.63; 95% CI: 1.42–4.86). Subjects reporting any alcohol use had a 68% higher prevalence of reporting risky sex compared to those subjects not reporting alcohol use (uPR = 1.68; 95% CI: 1.38–2.05).

The likelihood of being sexually active increased with increasing severity of alcohol use with the unadjusted prevalence ratio of sexual activity increasing from 1.98 among subjects reporting any alcohol use to 2.36 among subjects reporting alcohol misuse to 4.15 among subjects reporting hazardous alcohol use. Similarly, the unadjusted prevalence ratio of reporting any risk sex increased from 1.70 among those reporting any alcohol use to 1.90 among subjects misusing alcohol to 1.93 among subjects with hazardous alcohol use while the unadjusted prevalence ratio of reporting high risk sex was 1.44, 2.67, and 2.60 among subjects reporting any alcohol use, alcohol misuse and hazardous alcohol use, respectively.

Results for gender stratified univariable and multivariable analyses of factors associated with risky sex are shown in Table 3. Among males, ART receipt, alcohol misuse, HIV disclosure status, religious affiliation and adherence status were associated with risky sex in univariable analyses and therefore selected for the initial multivariable model. In the final multivariable model among males, reporting any risky sexual behavior was independently associated with alcohol misuse (aPR = 1.67; 95% CI: 1.07–2.60, *p* = 0.023), not disclosing HIV status to sexual partner (aPR = 1.69; 95% CI: 1.11–2.58, *p* = 0.014), being non-adherent to medication (aPR = 1.21; 95% CI: 0.98–1.50, *p* = 0.080), while controlling religious affiliation. Although subjects’ religious affiliation was not statistically significant variable in the final model, it was retained in the final model because it improved model fit with a significantly lower model QIC-U.

**Table 3:** Factors associated with reporting risky sexual behavior among 74 men and 61 women attending the Infectious Diseases Institute HIV Clinic, Kampala, Uganda.

Characteristic	Males				Females			
	UnadjustedPR (95% CI)	*p*	AdjustedPR (95% CI)	*p*	UnadjustedPR (95% CI)	*p*	AdjustedPR (95% CI)	*p*
Reported alcohol misuse	1.50 (1.00–2.24)	0.048	1.67 (1.07–2.60)	0.023	1.47 (1.09–1.99)	0.011	1.29 (0.95–1.74)	0.098
Did not disclose HIV status to sexual partner (vs. disclosed)	1.54 (1.00–2.37)	0.049	1.69 (1.11–2.58)	0.014	2.47 (1.87–3.27)	<0.001	2.45 (1.85–3.23)	<0.001
Non-adherent to medication^1^	1.31 (1.06–1.62)	0.012	1.21 (0.98–1.50)	0.080	1.46 (1.05–2.03)	0.024	1.42 (1.02–1.97)	0.036
Religion^2^								
Anglican	0.99 (0.75–1.30)	0.945	0.84 (0.62–1.13)	0.470	0.87 (0.63–1.21)	0.407	NS	
Roman Catholic	0.90 (0.68–1.20)	0.491	0.91 (0.69–1.18)	0.249	0.83 (0.59–1.17)	0.289	NS	
Receiving ART (yes vs. no)	0.82 (0.64–1.04)	0.094	NS		0.83 (0.61–1.13)	0.233		
Education level (vs. tertiary/diploma)								
Secondary level	1.24 (0.88–1.74)	0.213	-		0.78 (0.46–1.32)	0.359	-	
Primary or less	1.12 (0.78–1.59)	0.541	-		0.80 (0.48–1.33)	0.384	-	
Marital status (vs. married or cohabiting)								
Never married	0.89 (0.57–1.40)	0.626	-		0.83 (0.57–1.20)	0.315	NS	
Separated/widowed	0.90 (0.67–1.21)	0.475	-		0.72 (0.53–0.99)	0.043	NS	
Paid Employment (no vs. yes)	0.98 (0.63–1.50)	0.911	-		0.89 (0.60–1.34)	0.586	-	
Income in last month (vs. declined to answer)								
<20 USD	0.80 (0.45–1.44)	0.459	-		1.21 (0.51–2.87)	0.661	NS	
20–400 USD	1.14 (0.78–1.68)	0.501	-		1.38 (0.59–3.23)	0.453	NS	
>400 USD	0.90 (0.41–1.97)	0.789	-		3.50 (1.53–8.01)	0.003	NS	

NS = not significant

^1^ Medications included ART and/or co-trimoxazole. Non-adherent implied ≤95% score on a Visual Analogue Scale.

^2^ Reference religions include those that prohibit alcohol consumption: Pentecostal Christianity, Islam, and Seventh Day Adventism. Adjusting for religion among males provided model fit therefore left in the final model.

Among females, the univariable analyses (Table 3) identified alcohol misuse, HIV disclosure adherence, marital status and income in last month as variables eligible for the initial multivariable model. In the final multivariable model for females, reporting any risky sexual behaviors was independently associated with non-disclosure of HIV status (aPR 2.45; 95% CI: 1.85–3.23, *p*<0.001) and being non-adherent to medication (aPR 1.42; 95% CI: 1.02–1.97, *p* = 0.036), but not significantly associated with alcohol misuse (aPR 1.29; 95% CI: 0.95–1.74, *p* = 0.098).

## Discussion

In a large HIV clinic located in a typical urban setting in Sub-Saharan Africa, one in every three subjects receiving HIV care reported alcohol consumption within the previous 6 months. Alcohol misuse and hazardous alcohol use was reported by 19% and 6% of subjects, respectively, while 15% of drinkers reported at least one episode of binge drinking in the last 6 months. These finding are consistent with results from studies in other HIV clinic populations in Uganda [32,33], and some clinical settings in North America [34]. However, the prevalence of any alcohol consumption was relatively lower compared to studies done in the general medical settings [35] and general community populations [36,37] and far lower than those from fishing communities around Lake Victoria in central Uganda which reported that 62% of male and 52% of female had drank alcohol during the last month [38]. The relatively lower prevalence of alcohol use among patients in long-term HIV care, compared to the general population, may be a result of continued counseling and risk behavior change messages including reducing alcohol consumption. Despite this, the proportion reporting alcohol consumption in the clinic is half that of the general population in Uganda [39], while the prevalence of unprotected sex reported in this study is consistent with studies in sub-Saharan Africa [7,40].

We observed that subjects receiving ART were less likely to consume alcohol than subjects not yet on ART. This differential report of alcohol use by ART use status could be due to the intensive pre-ART initiation counseling sessions that includes adherence support, HIV disclosure, sexual risk, alcohol, drug and smoking reduction messages [41]. Another explanation could be that subjects who are on ART have previously experienced an advanced HIV disease or were at one time sicker and so stopped drinking due to illness while those not yet on ART are most likely still much healthier, likely not to have been seriously ill and therefore did not stop their alcohol consumption. This is in line with another longitudinal study that demonstrated a significant decline in reported alcohol use after ART commencement [33].

An unusual finding in this study was that females with low CESD scores, therefore less likely to have depression, were twice as likely to report alcohol misuse as compared to their counterparts with high CESD scores. It is possible that individuals with depression and higher alcohol consumption rarely enter HIV care or are quickly lost to follow-up, resulting in a study population yielding the paradoxical findings in our study. It is plausible that our study recruited mainly subjects without depression for they tend to be retained in care as compared to those with depression who tend to fall out of care earlier on [42,43].

Males were more likely than females to report alcohol consumption, misusing alcohol, and engaging in risky sexual behavior. The higher consumption among males has been reported in most studies both in the Sub-Saharan Africa and from developed countries in North America and Europe and are attributed to the acceptance of male drinking as part of the social values and attributes of masculine norms while at the same time barring this among women [44]. Although many studies have previously analyzed alcohol use data combined for both men and women, our study approach of analyzing this data by gender stratum was informed by previously published work showing that alcohol use and sexual behavior associations in men and women represents quite differential effects, and appears to be more complex in females than males as it involves both her own alcohol consumption, alcohol consumption of her male partner and the interaction with her male partner [45]. Published literature further shows that, risky sexual behavior among females depends heavily on the male partners behaviors such as condom use, alcohol use status and the gendered power dynamics in that sexual relationship [46,47]. Kalichman and colleagues, demonstrated that the likelihood of engaging in unprotected sex among males in southern Africa depended on both the male’s and the female partner’s alcohol consumption while among females; only their male partner’s consumption was significantly associated with the same risk [12].

Although there were marked gender differences in the degree of association, overall, this study has shown that subjects who consumed alcohol were more likely to report sexual intercourse, more likely to report risky sex in general and more likely to specifically engage in unprotected sexual intercourse. Woolf King *et al* basing on theoretical considerations and empirical research proposes two gender specific models to explain this association through the transmission hypothesis as applicable to Sub-Saharan Africa [22]. In general, both models proposed posit that the higher the alcohol consumed, the more likely is the person consuming alcohol to experience the psychoactive myopic effects and the psychological expectancy effects which propagate the reduced fear of negative consequence of engaging in risky sexual behaviors resulting in increased likelihood of engaging in risky sexual behaviors. The latter could result in further transmission of HIV (and other sexually transmitted infections) if all other conditions necessary for transmission exist.

In addition, subjects reporting alcohol misuse were also more likely to be non-adherent to medication (both co-trimoxazole and ART) and more likely not to disclose their HIV positive status to their sexual partner(s), all of which are behaviors that partly negatively impact on overall success of ART. The co-occurrence of these behaviors has been highlighted previously in rural Uganda [16,48] and we postulate that a pattern of risk prone, sensation seeking, personality of individuals drives them to seek comfort and sensation by consuming alcohol [49].

Therefore, interventions to reduce alcohol consumption may reduce the co-occurrence of the associated harmful behaviors. Prevention messages designed to encourage reduction in alcohol use among PLWHA should also support HIV disclosure and medication adherence efforts. These prevention messages should be target men and women separately [11].

Subjects in our study who belonged to religious denominations (Pentecostals, Muslims and Seventh-Day Adventists) that strongly prohibit and openly preach against alcohol consumption were less likely to consume alcohol, an observation found elsewhere [50]. Although, in part, this may be due reporting bias, we propose that clinic based interventions incorporate patients’ religious belief, if relevant, as an entry point to further tackle alcohol consumption reduction, as had been observed in a community setting in Uganda [38].

There is a rationale for strengthening routine positive prevention counseling messages among those not yet receiving ART. Although these individuals, on average, are indicated to attend HIV clinic with less frequency, we observed that they were more likely to report both alcohol consumption and risky sexual behaviors.

The findings of this study need to be examined within the context of the several limitations. First, all behaviors were obtained by self-report and therefore prone to both social desirability and recall bias. However any of the biases, if present, are not as likely to alter the overall results because these results represent the least likely estimates of alcohol use given the tendency for individuals to underreport rather than over report alcohol consumption. However, we restricted our inquiry to the last 6 months (as opposed to one year) to reduce on the likelihood of recall bias. Furthermore, any associations between alcohol and any other behavior described here are based on the global associations rather than event level measurement therefore minimizing the bias. We believe that our findings are internally valid because of the rather expected differential alcohol consumption reported among females and males and the higher risky behaviors reported in males as compared to females, both of which are in line with known gender differences [27]. Given the cross-sectional design of this study, it is difficult to know whether alcohol use preceded risky sexual behaviors or the reverse. It is possible some common personality traits makes people more likely to engage in multiple risky behaviors including drinking alcohol and all these are manifestations of this risky personality trait [51]. Another limitation is the possibility of reverse causality: those who engage in risky sexual behavior may choose to drink alcohol to engage in this behavior more effectively. Finally, the absence of data on duration of HIV care limited us from exploring the effect of duration of treatment/care on reported behaviors.

The strengths of this study include the relatively large number of subjects interviewed, use of the AUDIT, a standardized internationally-validated tool for alcohol assessment in primary care settings, allowing for cross-study comparability. Furthermore, unlike previous studies that have focused on more rural or peri-urban areas, this study examines alcohol consumption among PLWHA living within a major urban city in sub-Saharan Africa.

Although this study looked only at the behavioral associations of alcohol use and risky sexual behaviors, it is important to contextualize that alcohol plays a bigger role in the overall HIV transmission and HIV disease progression risk through its effect on transmission dynamics, viral replication, host immunity responses and its impact on ART efficacy [52]. We believe there is a rationale for future studies to test the efficacy of brief interventions to reduce alcohol consumption among PLWHA. These interventions should be based on locally appropriate messages and would need to address the constellation of other negative behaviors that are exhibited by persons who consume alcohol while in long term HIV care. Brief interventions could be delivered in conjunction with pre-ART counseling, and could incorporate motivational interviewing techniques [53]. Given the shortage of healthcare workers in this setting, for pragmatic reasons, brief interventions for alcohol use would need to be conducted by routine clinic personnel or paraprofessional personnel [54].
